# CCAST: A Model-Based Gating Strategy to Isolate Homogeneous Subpopulations in a Heterogeneous Population of Single Cells

**DOI:** 10.1371/journal.pcbi.1003664

**Published:** 2014-07-31

**Authors:** Benedict Anchang, Mary T. Do, Xi Zhao, Sylvia K. Plevritis

**Affiliations:** Department of Radiology, Center for Cancer Systems Biology, Stanford University, Stanford, California, United States of America; University of California, San Diego, United States of America

## Abstract

A model-based gating strategy is developed for sorting cells and analyzing populations of single cells. The strategy, named CCAST, for Clustering, Classification and Sorting Tree, identifies a gating strategy for isolating homogeneous subpopulations from a heterogeneous population of single cells using a data-derived decision tree representation that can be applied to cell sorting. Because CCAST does not rely on expert knowledge, it removes human bias and variability when determining the gating strategy. It combines any clustering algorithm with silhouette measures to identify underlying homogeneous subpopulations, then applies recursive partitioning techniques to generate a decision tree that defines the gating strategy. CCAST produces an optimal strategy for cell sorting by automating the selection of gating markers, the corresponding gating thresholds and gating sequence; all of these parameters are typically manually defined. Even though CCAST is optimized for cell sorting, it can be applied for the identification and analysis of homogeneous subpopulations among heterogeneous single cell data. We apply CCAST on single cell data from both breast cancer cell lines and normal human bone marrow. On the SUM159 breast cancer cell line data, CCAST indicates at least five distinct cell states based on two surface markers (CD24 and EPCAM) and provides a gating sorting strategy that produces more homogeneous subpopulations than previously reported. When applied to normal bone marrow data, CCAST reveals an efficient strategy for gating T-cells without prior knowledge of the major T-cell subtypes and the markers that best define them. On the normal bone marrow data, CCAST also reveals two major mature B-cell subtypes, namely CD123+ and CD123- cells, which were not revealed by manual gating but show distinct intracellular signaling responses. More generally, the CCAST framework could be used on other biological and non-biological high dimensional data types that are mixtures of unknown homogeneous subpopulations.

## Author Summary

Sorting out homogenous subpopulations in a heterogeneous population of single cells enables downstream characterization of specific cell types, such as cell-type specific genomic profiling. This study proposes a data-driven gating strategy, CCAST, for sorting out homogeneous subpopulations from a heterogeneous population of single cells without relying on expert knowledge thereby removing human bias and variability. In a fully automated manner, CCAST identifies the relevant gating markers, gating hierarchy and partitions that isolate homogeneous cell subpopulations. CCAST is optimized for cell sorting but can be applied to the identification and analysis of homogeneous subpopulations. CCAST is shown to identify more homogeneous breast cancer subpopulations in SUM159 compared to prior sorting strategies. When applied to normal bone marrow single cell data, CCAST proposes an efficient strategy for gating out T-cells without relying on expert knowledge; on B-cells, it reveals a new characterization of mature B-cell subtypes not revealed by manual gating.

## Introduction

Understanding cancer heterogeneity is increasingly being regarded as critical in understanding cancer progression and overcoming therapeutic resistance [1]–[4]. Different types of heterogeneity are commonly observed among the cells composing a single tumor, including genetic [5], [6], epigenetic [7], and phenotypic heterogeneity [3], [4]. Although technological challenges have limited our ability to fully characterize intra-tumor heterogeneity, in recent years characterizing heterogeneous populations of cells at the single-cell level using multidimensional fluorescence and mass flow cytometric data, combined with novel computational tools, has greatly improved our understanding of the extent of cellular heterogeneity [8], [9]. Moreover, by sorting out homogeneous subpopulations, researchers can measure and compare genomic and other functional properties of different subpopulations. However, in spite the high-throughput nature of these single cell measurements, current methods for sorting specific cell subpopulations rely on a low dimensional, often user-defined, process known as gating. Gating on a fluorescence-activated cell sorting (FACS) machine commonly refers to a manual process, performed by sequentially selecting regions from bivariate graphs that depict the expression of two markers at a time across all the cells. The gating strategy often relies on an expert's assessment of the choice of gating markers, the order of gating and cut points to identify each gated region; this assessment is often based on a subjective analysis using packages such as flowJo and FlowCore [10]. It is well documented that minor differences in gating strategy can lead to significantly different quantitative conclusions [11], [12]. We present a gating strategy that is optimized for cell sorting. Because our gating strategy is data derived, we argue that is optimal compared to manually-derived gating strategy which can be biased and highly variable. In our work, we make a distinction between gating algorithms that are optimized for sorting single cells versus analyzing a heterogeneous population of single cell data. Even though our gating strategy is optimized for cell sorting, it also has value when used in analysis of population data at the single cell level.

When analyzing a population of single cells, several gating algorithms have been developed to reduce the technical, biological and human sources of variation involved in identifying and analyzing clusters of similar cell subpopulations [8], [9], [13]–[17]. Bashashati and Brinkman provide a comprehensive overview of analysis tools for flow cytometry (FCM) data [18]. More recently, the FlowCAP-II project [12] compared the accuracy and reproducibility across several gating algorithms in terms of identifying cell clusters. All gating algorithms, including ours, have some form of a clustering algorithm, which is used to identify homogeneous subpopulations, as a major component. Many unsupervised clustering algorithms take into account the uncertainty in cluster assignments by modeling the data as mixtures of parametric distributions [18]. Although parametric mixture models have been developed to analyze FCM data [14], computational, as well as estimation errors, in clustering could still arise from outliers and skewness in the data which may not reflect the underlying assumptions of the parametric model. As an alternative, we propose a modified version of the non-parametric multivariate mixture modeling approach by Benaglia *et al.* [19] for clustering FCM data, where our modification includes the use of silhouette measures. This clustering algorithm handles uncertainty regarding to which cluster an event should be assigned as well as the uncertainty in the number of underlying cell states in the heterogeneous parent population and makes little or no prior assumptions on the underlying model structure. In addition, we implement an alternative clustering algorithm, namely hierarchical clustering [20], to show that the results from our gating strategy are independent of the particular clustering method used. The goal of our study is not to provide an optimal clustering strategy, but instead to provide an optimal gating strategy for sorting homogeneous cell subpopulations given any reasonable clustering algorithm.

A commonly neglected area in studying populations of single cells is identifying an optimal gating strategy for cell sorting. Sorting cells for downstream analysis relies not only on the identifying the clusters but also on the gating strategy, which is defined by the gating markers, thresholds and sequence. For manual gating at the FACS machine, typical gating strategies are organized like a family tree. For example, from mature bone marrow cells, lymphocytes are gated from the parent cells and from that gate, T-cells or B-cells are gated, and from those gates, specific T-cell and B-cell types are gated [9]. In particular, sorting out T-cells is equivalent to isolating a CD4+/CD8+ population; the user would first isolate the lymphocytes, then derive the CD3+ cells and from there, would draw a gate around the CD4 positive and CD8 positive subpopulations. This approach assumes prior knowledge of the underlying set of markers that define cell types, the gating hierarchy and relative boundaries for isolating pure cell subpopulations of interest. Selecting these parameters based solely on literature and human perspective introduces bias and variability and could result in contamination among the cell subpopulations. We make this process data-driven and fully automated by applying a recursive partitioning technique that generates a decision tree representing a reproducible gating strategy for all subpopulations of interest.

Recognizing the current reliance on human perspective and intuition in manual gating, Ray and Pyne [17] recently developed a gating framework which emulates the human perspective in FCM data analysis based on a mathematical map of the high dimensional data landscape. They propose flexible, sample-specific templates for extracting features of interest, which may have unusual shapes and distributions. An alternative approach by Lee *et al* [21] uses transfer learning technique combined with the low-density separation principle; this approach transfers expert knowledge on training FCM data sets to a new data. A more recent study by Aghaeepour *et al.* [22] developed a supervised learning computational framework that automatically reveals cell subsets that correlate strongly with clinical outcome and identifies their relevant set of markers for gating. In a follow-up study, Aghaeepour *et al.* [22] developed a computational tool, RchOptimyx [23], that uses dynamic programming and optimization techniques from graph theory to construct a cellular hierarchy, providing a gating strategy to identify target populations to a desired level of purity. One might argue that our work is most similar to RchOptimyx. However, as will be shown later, RchyOptimyx provides multiple approaches for gating a specific subpopulation, whereas our approach aims to find a single, optimal gating strategy in a fully automated manner without relying on qualitative judgement.

We present an algorithm, named CCAST for Clustering, Classification and Sorting Tree, to identify and sort homogeneous subpopulations from a heterogeneous parent population using a decision tree representation for a gating strategy that can be used to sort for homogeneous cell subpopulations. The gating strategy derived from CCAST is data-driven and fully automated and it does not rely on expert knowledge. While CCAST is optimized for cell sorting, CCAST also has value when applied to data analysis by filtering and retraining the decision tree to produce more homogeneous subpopulations. In addition, when used for data analysis, CCAST may identify new subpopulations among the initial clusters. We apply CCAST on populations of single cell measurements made on breast cancer and normal human bone marrow. On the breast cancer SUM159 cell line, CCAST reveals at least 5 distinct cell states based on two surface markers (CD24 and EPCAM). When applied to normal bone marrow data, CCAST reveals an efficient strategy for gating T-cells. In addition, CCAST reveals two new mature B-cell subtypes, which were not found by manual gating but show distinct intracellular signaling behaviors.

## Results

**Figure 1:**
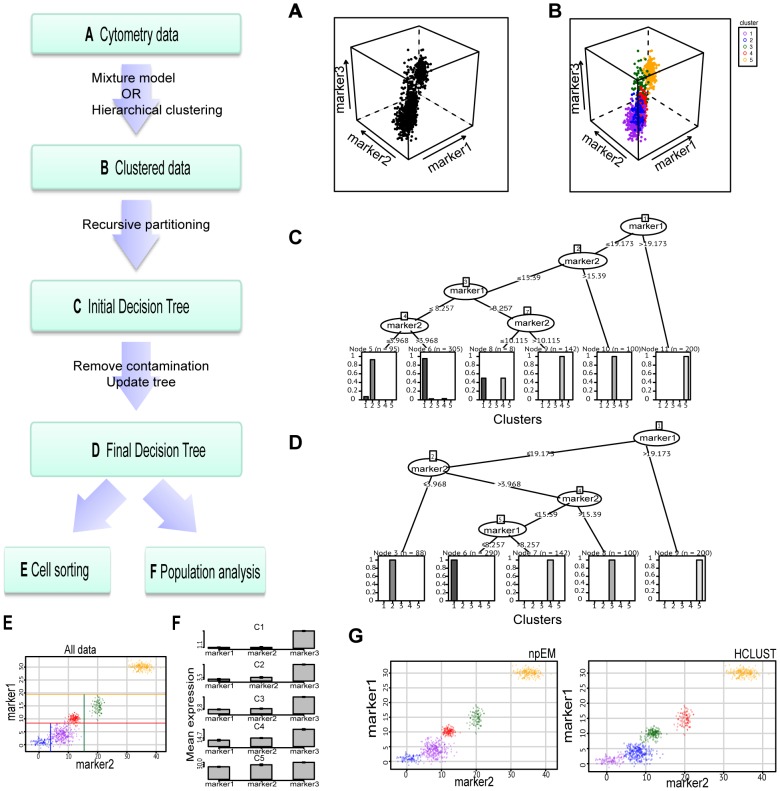
CCAST flowchart and analysis on a simulated dataset. **A** Cytometry data represented by 3D scatterplot of simulated FCM data showing the expression of 3 markers across all cells. **B** Clustering analysis produces five cell types color coded and denoted as Cell-types 1, 2, 3, 4 and 5. **C** Initial CCAST decision tree generated showing subpopulations at the leaf nodes. Nodes 9, 10 and 11 contain a single cell type and are considered as pure subpopulations. Nodes 5 6 and 8 contain a mixture of cell types. **D** Final CCAST decision tree obtained after filtering the data by removing contaminating cells in nodes with mixed cell-types. This tree can be used for cell sorting or data analysis. **E** 2D scatter plot of original (unfiltered) data showing the 5 clusters color coded and estimated cut-offs with corresponding color-coded thresholds for sorting the 5 cell state populations. Note that the subpopulations can be sorted using only Marker 1 and Marker 2 even though three markers were initially used to identify the cell types. **F** Bar plot the three markers in each subpopulation derived using the final CCAST tree on the filtered data. **G** 2D scatter plot of the filtered data derived from CCAST showing the analysis derived from hierarchical (right) versus npEM (left) clustering are similar.

We demonstrate the performance of CCAST on simulated and actual populations of single cell data. The details of the CCAST algorithm are provided under Materials and Methods. In Figure 1, CCAST is summarized in a flowchart alongside its application to simulated data. Briefly stated, starting with a population of single cell data (Figure 1A), CCAST performs a cell clustering algorithm to identify groups of similar cells (Figure 1B). The clustering can be performed in a variety of ways. We implement a nonparametric mixture model denoted as “npEM” (see Materials and Methods), but show that other clustering algorithms, such as hierarchical clustering (HCLUST), produces similar gating strategies within the CCAST framework. Once the cell clusters (aka “cell types”) are established, CCAST derives a gating strategy that is represented by a decision tree (Figure 1C), where the nodes specify the gating markers and their thresholds (aka “split points”) as edges. The terminal leaves of the decision tree represent the final gated subpopulations. Often the final number of gated populations is greater than the number of cell clusters. When this happens some of the subpopulations capture cells from only one cluster, but others capture cells from multiple clusters. For the subpopulations which contain cells from multiple clusters, all but the cells from the dominant cluster are removed and CCAST is retrained on the remaining population, producing a more robust gating strategy because it is less influenced by “contaminating” cells (Figure 1D). The final decision tree can be used for cell sorting (Figure 1E) or data analysis (Figure 1F). Although not shown in Figure 1, it is possible that a single cluster may be distributed across multiple subpopulations, where each subpopulation only contains cells from that cluster; in those cases, the cluster may have more subpopulations than derived by the clustering algorithm. This feature and all other mentioned features of CCAST are demonstrated below.

We implemented CCAST as an R package and it has been made available as a zip file in the Supplement.

### CCAST is robust to the clustering algorithm, as evidenced on simulated data

To illustrate the basic properties of CCAST, we applied it to a simulated dataset of 850 single cells comprised of a mixture of 5 cell types, as illustrated in Figure 1A. On each single cell, 3 markers are measured; the distributions of marker values for each cell type are summarized in Supplement Table S1. We sampled 100, 300, 150, 100, 200 cell vector expression values for each cell type respectively. Figure 1B shows the 3D scatter plot of the cell measurements with the 5 cell types color coded; from this figure, it is not automatically apparent how to optimally sort out these 5 clusters. Figure 1C shows the first CCAST-derived decision tree based on the entire dataset; this tree partitioned the data into 5 clusters as evidenced from the leaf nodes (5,6,9,10 and 11) of the tree. Nodes 9, 10 and 11 represents pure subpopulations of clusters 4, 3 and 5, respectively; node 8 shows a mixture of clusters 1 and 4; nodes 5 and 6 are dominated by cells from clusters 2 and 1 respectively. After CCAST removed the contaminating cells from the subpopulations that have more than one cluster and re-ran the decision tree algorithm, it generated the final decision tree in Figure 1D. Note also that these subpopulations were gated using only two markers even though 3 markers were measured. Figure 1E shows the application of the final decision tree (Figure 1D) on the entire dataset. When this gating strategy was applied to the filtered dataset for downstream analysis, the resulting subpopulations are shown in Figure 1F, represented with bar plots of the markers' expression and labeled by their corresponding cell cluster. Figure 1G shows the application of the gating strategy using the estimated cut-offs on the entire data using hierarchical clustering instead of “npEM” clustering. The similar partitions on the 2D data imply that using different a clustering algorithm results in similar homogeneous subpopulations.

### CCAST is reproducible, as evidenced on T-cell mass cytometry data

We next demonstrate the applicability of CCAST on actual hematopoietic dataset obtained in the study by Bendall *et al.* [9]. This study analyzed normal bone marrow at the level of single cells using mass cytometry (MCM), which is a recently developed high throughput technology for labeling single cells with metal-chelated antibodies that reduce auto fluorescence effect. An appeal of this particular study is that hematopoietic cells have a well-established set of lineage markers defining their differentiation stages. In this study, unstimulated and stimulated human peripheral blood mononuclear cells (PBMCs) from a healthy donor were analyzed using thirteen surface parameters, namely: CD45, CD45RA, CD19, CD11b, CD4, CD8, CD34, CD20, CD33, CD123, CD38, CD90, and CD3. In addition, 18 intracellular signaling molecules were measured. The manual gating process and the characterization of the major cell populations are shown in Supplement Figure S5 of [9]. One part of this study focused on a T-cell subset that included naive CD4+ and CD8+ T-cells and mature CD4+ and CD8+ T-cells. The analysis of the induced intracellular signaling responses in these subpopulations, as compared with those of an unstimulated control, relied on a manually-defined gating process.

**Figure 2:**
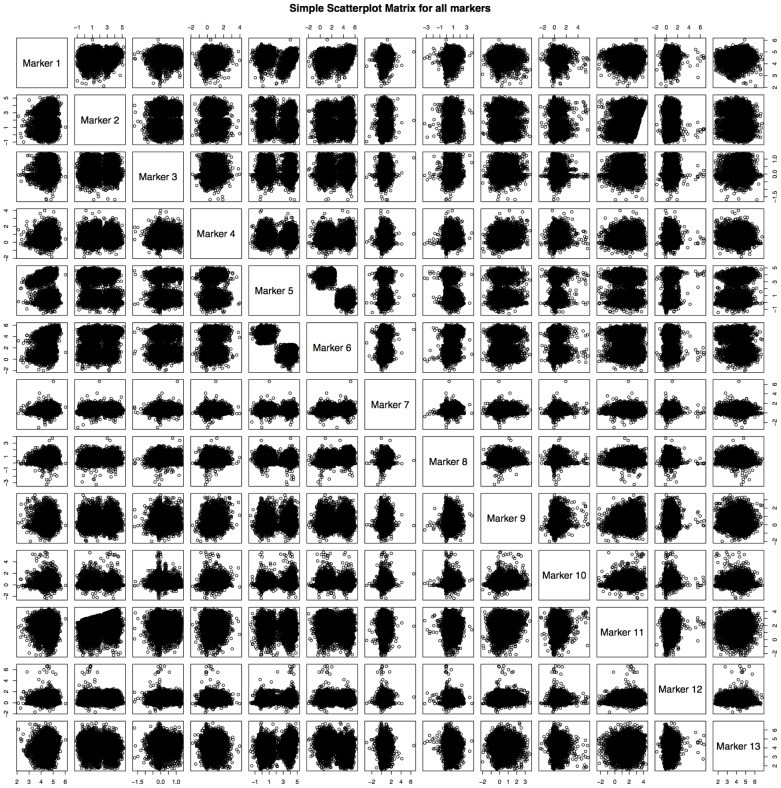
Visualization of 13 markers across heterogeneous population of T-cells. These 13×13 scatter plots show pair-wise distribution of 13 markers (unlabeled) per cell from pooled single cell data of 4 T-cell subtypes. Primary data was made publicly available by Bendall *et al.* [9].

**Figure 3:**
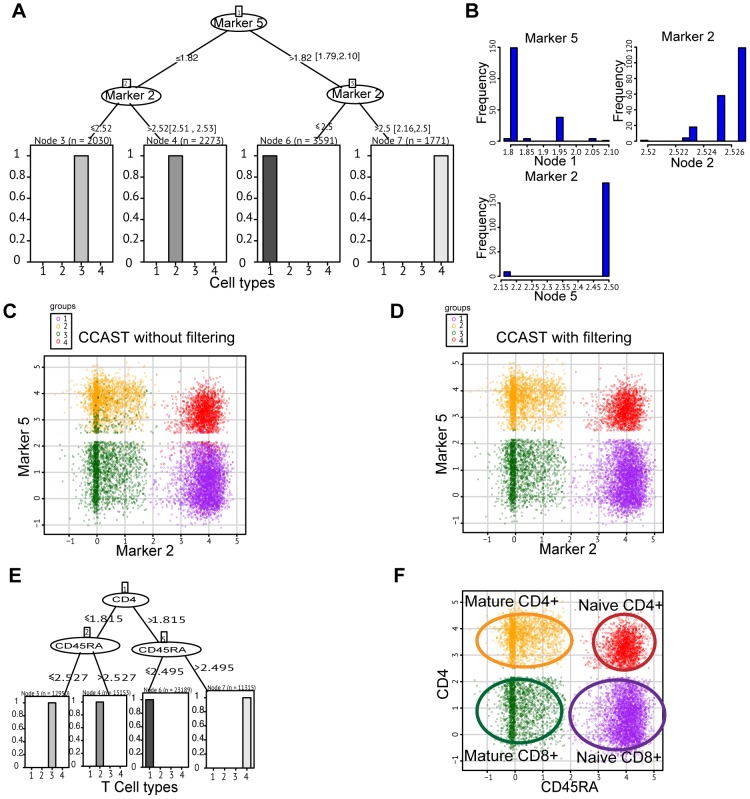
CCAST applied to single cell analysis of T-cells. **A** The CCAST gating strategy based on the unlabeled T-cell data in Figure 2, post filtering, showing that 4 cell types can be derived using only Marker 5 and Marker 2 with Marker 5 as the root node. Split points along with the minimum-maximum range for each split point are provided at each node. **B** Histogram plots for sample split point for each node is obtained via bootstrapping. The multi modal nature of the distributions makes it difficult to calculate a true confidence intervals on the split point estimates. **C** CCAST result without filtering represented as a 2D scatter plot of the 4 cell types, which each cell type color coded; note that gating the yellow-colored cells will likely result in contamination of green-colored cells. **D** CCAST result with filtering represented as a 2D scatter plot of the 4 pure cell types, with each cell type color coded. Note all contaminating cells mixed with various clusters have been removed. For manual gating purposes, comparing the two schemes **C** and **D** provides a visual evaluation of the expected contamination levels from sorting subpopulations. **E** CCAST gating strategy for all Tcell types with labels reveals that the key gating markers are CD4 and CD45RA markers. **F** 2D scatter plot for the four, labeled T-cell types based on CD4 and CD45RA.

To demonstrate the robustness of CCAST, we consider a subset of the data from the study by Bendall *et al.* [9] in order to assess both the error and reproducibility of our results in a transparent manner. We focus on a 20,000 cell T-cell subpopulation which had been manually gated into 4 subtypes (see Figure 1 in Bendall *et al.* [9] for the manual gating scheme). Here we pool this manually gated T-cell data, then blind the data by removing all prior knowledge of cell types or marker labels. We then randomly separate this data into a training and test set of 10,000 cells each. Pairwise scatter plots across all 13 markers, unlabeled, are shown in Figure 2. We apply CCAST on the training data to obtain the final decision tree shown in Figure 3A. These results indicate that the 4 distinct homogeneous cell states can easily be isolated using only 2 of the 13 measured markers, namely Marker 5 and Marker 2. We next carried out a sensitivity analysis on the decision tree parameters, namely the optimal tree height, denoted as L, and the split points (see Materials and Methods). First we ask the question: what happens to the purity of the homogeneous subgroups if we increase the level of pruning the decision tree, L? Figure S1A in the supplement document shows exactly the same decision tree as in Figure 3A after increasing L to 3 or more levels. In fact, an L-sensitivity analysis with the simulated 3D data (described above) showed that increasing L above 4 produces the expected 5 homogeneous groups but decreases the expected number of cells per group (results not shown). CCAST automatically determines L based on the homogeneity of the subpopulations (Materials and Methods). Next, we performed a bootstrap analysis to assess the range of values for the split points in the optimal decision tree. More specifically, we performed a strata-sampling method with replacement to generate 200 bootstrap datasets of the same sample size as the training data. We ran CCAST on these samples to generate 200 decision trees with different split points. The hierarchy and selected markers for these bootstrap samples were exactly the same as shown in Figure 3A. We show the confidence intervals of the split points by minimum and maximum boundary estimates from our bootstrap analysis (see range located beside split point estimates in Figure 3A). Note that we could not calculate the normal confidence intervals for these split point estimates due to the multi-modal nature of the split point distributions (Figure 3B). To test the performance of CCAST, we applied CCAST on the test data using decision tree derived from the training set (Figure 3A). After data filtering, the final decision tree on the test dataset is shown in Supplemental Figure S1B. Note that all split point estimates lie within the previously estimated confidence intervals shown in Figure 3A. In addition the hierarchy of the tree remains the same. This result demonstrates that CCAST yields robust split point estimates and can produce reproducible results. Finally, we compare the CCAST result before (Figure 3C) and after data filtering (Figure 3D). Figure 3C show a 2D scatter plot of the 2 markers that partition the training data into clearly 4 clusters. Although there is a strong evidence of 4 clusters, it is apparent that sorting out the population in the yellow cluster without contaminating green cells would be challenging. Figure 3D show the results after applying CCAST on the training data for data analysis. Notice the pure subpopulations after applying the data-filtering step of CCAST. Hence, in addition to providing a gating strategy, CCAST can also produce a more homogenous representation of the original data for data analysis.

### CCAST provides an efficient gating strategy for T-cells

Using the T-cell dataset described above, we show that our CCAST-derived gating strategy reproduces the manual gating results in Bendall *et al.* [9] without relying on expert knowledge. Figure 3E shows that CCAST isolates the 4 distinct T-cell states using only 2 of the 13 measured surfaces markers. These two markers turn out to be CD4 and CD45RA. Figure 3F shows the distribution of the 4 labeled T-cell subtypes based on CD4 and CD45A expression. This result demonstrates that CCAST can identify the 2 of 13 markers that are known to be most relevant to identifying the subtypes of interest without relying on prior knowledge of the subtypes or the markers that are best known to define them. Moreover, for data analysis, CCAST provides more homogeneous subpopulations by filtering out the contaminating cells; an analogous step was not performed in the manually gated analysis [9].

### CCAST reveals additional B-cell subtypes in pooled manually gated subpopulations

**Figure 4:**
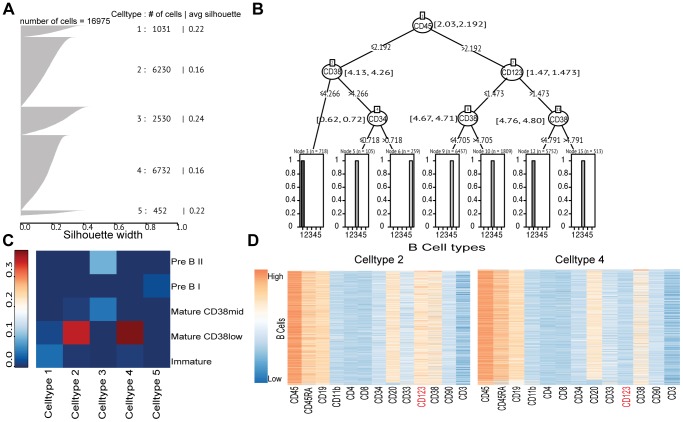
CCAST applied to single cell analysis of B-cells. **A** Silhouette plot showing evidence of 5 B-cell types. **B** CCAST gating strategy for B-cell types based on CD45, CD34, CD38, and CD123 markers using 3 levels of gating. The estimated ranges for the split point variables are provided at each node. Note Celltype 3 is distributed across three gated populations. **C** Cross classification heatmap of manually gated and CCAST predicted B-cell types indicates strong evidence that the most abundant Mature CD38low B-cells comprise a mixture of other subtypes (Celltype 2 and 4). **D** Heatmaps show evidence of the two derived distinct mature B-cell states corresponding to Celltypes 2 and 4 based mainly on CD123 (label highlighted in red).

**Figure 5:**
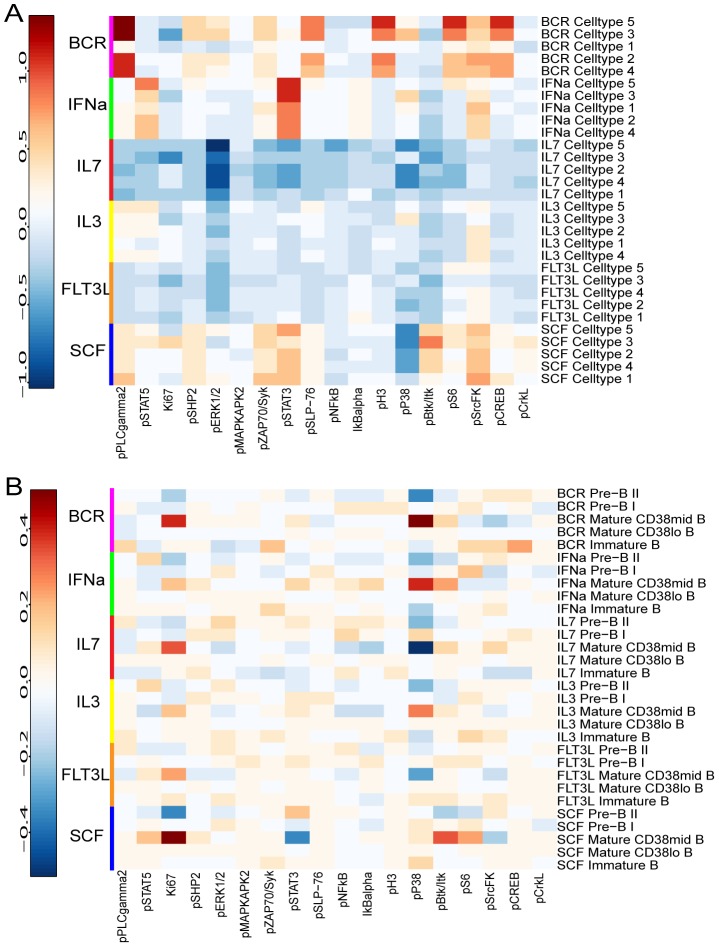
Signaling behavior in B-cell subtypes for CCAST vs manual gating strategy. **A** Heatmap of BCR, IFNa, FTL3, IL3, IL7, and SCF induced intracellular signaling responses in 5 B-cell CCAST-derived subtypes, compared with those of an unstimulated control. **B** Heatmap of BCR, IFNa, FTL3, IL3, IL7, and SCF induced intracellular signaling responses in the five B-cell subtypes obtained from the manual gates in Bendall *et al.* [9], compared with those of an unstimulated control. The higher difference implies a stronger signal in the CCAST-derived cell type compared to the manually gated cell type.

We next applied CCAST only on the manually gated B-cell subpopulations of the Bendall *et al.* study [9]. In this study, the manually gated B-cell subtypes were: early Pre-B I cells, late Pre-B II cells, immature B-cells, naive mature CD38mid B-cells and mature CD38low B-cells (see Figure 1 in Bendall *et al.* [9]). To verify the existence of these 5 major B-cell subpopulations, we performed hierarchical clustering, with a cutoff of 5 clusters, on the pooled manually-gated B-cell data, which consisted of about 17,000 cells. The silhouette plot in Figure 4A shows strong evidence of 5 clusters. Figure 4B shows the CCAST-derived gating strategy as a decision tree whereby the 5 distinct cell types can be isolated using only 4, of the 13, surface markers (namely CD45, CD34, CD38 and CD123) with only 3 levels of branching. A cross classification analysis between the CCAST-derived versus the manually gated subtypes is summarized as a heatmap in Figure 4C. Based on Figure 4C, we predict that subpopulations comprising CCAST-derived Cell-types 1, 4, 3, and 5 are predominately immature B, mature CD38low B, Pre B II, and Pre B I cells, respectively. However, there is not a clear one-to-one mapping between the CCAST-derived and manually gated subtypes. In particular, Figure 4C shows strong evidence of a mixture of the mature B-cell subtypes in CCAST Cell-types 2 and 4. The heatmaps in Figure 4D show evidence of two CCAST-derived distinct cell types corresponding to Cell-types 2 and 4 which were considered as one major population, namely mature CD38low B-cells, by manual gating. Based on surface marker expression, the most striking difference between Cell-types 2 and 4 is the expression of CD123, a signaling molecule which promotes proliferation and differentiation within the hematopoietic cell lines and is associated with hairy cell leukemia [24]. Figure 5A provides the heatmaps of BCR, IFNa, FTL3, IL3, IL7, and SCF induced intracellular signaling responses in the 5 CCAST-derived B-cell subtypes compared with an unstimulated control. For the purpose of comparing with the results of Bendall *et al.* [9] signaling induction was calculated using the difference of the mean scaled arcsinh value of unstimulated condition and the mean scaled arcsinh value of a stimulated condition; moreover, only the 13 surface markers were used to predict the cell types in the stimulated conditions using the decision tree from the unstimulated controls. The difference is calculated as a difference of absolute fold changes. BCR, IFNa, IL7 and SCF stimulations induce strong intracellular signaling across the B-cells across the different development stages. The heatmap in Figure 5B provides heatmaps of BCR, IFNa, FTL3, IL3, IL7, and SCF induced intracellular signaling responses for various B-cell subtypes derived from the manual gating in [9]. In the manually gated cells, the strongest signaling differences are limited to mature B-cells particularly associated with P38 and Ki67. In the CCAST-gated cells, BCR stimulation induces strong differences in PLC-gamma2 signaling, STAT3, H3, S6, CREB; IL7 stimulation alters ERK1/2 and P38 signaling, INFalpha alters STAT3 signaling; and SCF induces changes in P38 signaling. Overall, compared to the manually gated cell types, the CCAST-derived cell types exhibit more differences in stimulated induced signaling, presumably because the CCAST-gated subpopulations are more homogeneous. Finally, as an aside, we note that CCAST produces 7 homogeneously gated subpopulations, 3 of which belong to Cell-type 3, suggesting that this cell type may be more heterogeneous than suggested by the clustering algorithm.

### CCAST identifies at least five distinct cell types in SUM159 breast cancer cell line

**Figure 6:**
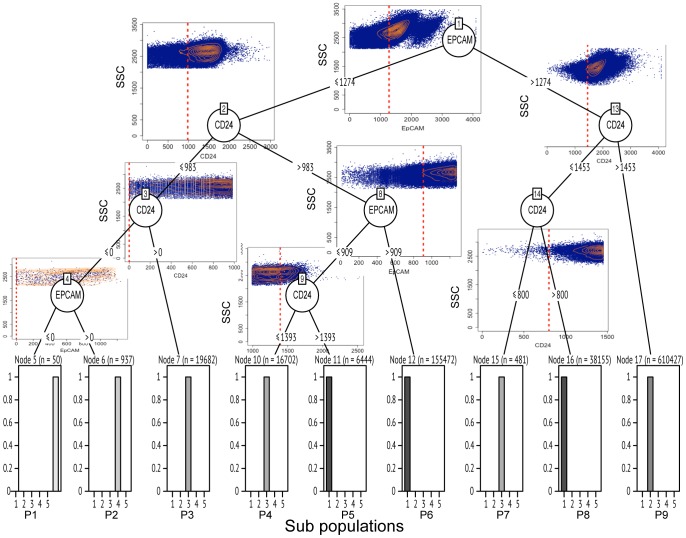
CCAST gating strategy on SUM159 breast cancer cell line. CCAST gating strategy for SUM159 breast cancer cell lines isolates 5 pure cell states (across 9 bins) based on CD24 and EPCAM. Visualization of these 5 subpopulations is clearly not apparent from the biaxial side scatter (SSC) vs. biomarker plots. Split point estimates (dotted red lines) go through density contour plot (orange) on the distributed data providing visual evidence for suitable cut-offs through bimodal contours. Note the split point lines for nodes 3 and 4 concentrate on the zero point mass; this indicates there are several cells with zero expression values for EPCAM or CD24 staining but with higher expression values with respect to CD44.

**Figure 7:**
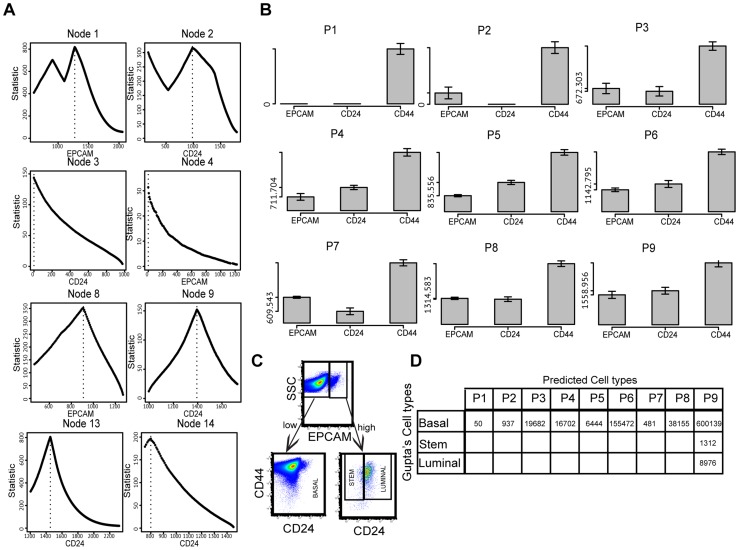
CCAST analysis on SUM159 breast cancer results. **A** Results for the estimation process for all the split point statistics in all the inner nodes in Figure 6. The root node corresponding to EPCAM shows one local maxima and one global maximum. Gating the data from this global maximum results in 9 distinct subpopulations. Nodes 3, 4, 8, 9, 13 and 14 have clear natural maxima indicating optimal splits for the data into these 9 homogenous subpopulations (see Figure 6) corresponding to the 9 bar plots in **B**. **B** Bar plots of the 9 homogenous subpopulations from Figure 6 across all 3 markers with standard deviation intervals for each marker. The values on the bars on the left side of each plot correspond to the minimum value for all 3 bar heights. Each side bar gives a sense of the relative difference between bar heights. The main title for each plot shows the corresponding leaf node bin on the tree in Figure 6. Predicted Celltypes 3 and 1 correspond to P3, P4, P7 and P5, P6, P8 respectively indicating more homogeneous sub populations than expected. The bar plots show evidence of at least 5 distinct sub populations i.e. P1, P2, P5, P7 and P9. **C** Gupta *et al.* [3] gating strategy isolated 3 cell states (Basal, stem, and luminal) using EPCAM as the major marker. They further use CD24 to sort out these 3 states. We also automatically identify EPCAM as the major marker but use a combination of multiple splits from CD24 and EPCAM to produce 9 homogeneous bins. **D** Comparison of predicted breast cancer subpopulations comparing the CCAST versus Gupta *et al.* [3] gating strategy shows potential evidence of contamination after sorting. This analysis indicated the CCAST subpopulation P9 is clearly a mixture of basal, stem, and luminal subpopulations from Gutpa *et al.* [3]. Unique CCAST subpopulations P1 and P2 were not even identified by Gupta *et al.* [3].

We applied CCAST on about 1 million cells of a SUM159 (triple negative) breast cancer cell line. We generated primary FACS analysis on SUM159 cell line for this study based on expression of EPCAM, CD24 and CD44 (see Materials and Methods). To assess the likely number of cell clusters in SUM159, we ran the “npEM” cluster algorithm, assuming 10 clusters, on a random subsample of about 3,000 cells and obtained 5 clusters. Using hierarchical clustering with a cut off of 5 clusters, on the entire SUM159 dataset, CCAST-derived the gating strategy that is shown in Figure 6. CCAST identified 9 homogenous subpopulations denoted as P1–P9 at the terminal nodes of the tree in Figure 6. A similar implementation on flowJo showing 9 homogeneous clusters is shown in Supplemental Figure S2. Figure 7A summarizes the results for the estimation process for all the split point statistics on all the inner nodes of the CCAST decision tree. The root node corresponding to EPCAM shows one global maximum indicating a strong split point. Nodes 3, 4, 8, 9, 13 and 14 have clear natural maxima indicating optimal splits for the data into clearly 9 subpopulations, each corresponding to 9 single mode histograms in the leaf nodes of the tree. Corresponding barplots for all 9 subpopulations with standard deviation intervals for each marker are shown in Figure 7B. A multivariate Hotelling's T square test showed significant differences between group pairs (p-value: 0), indicating that these 9 nine subpopulations are statistically different from each other. Interestingly, CCAST splits cluster 1 into the subpopulations P5, P6 and P8; it also splits cluster 3 into the subpopulations P3, P4 and P7.

Next we compare the results of the CCAST-derived gating strategy on SUM159 to the manually-defined gating strategy by Gupta *et al.* [3] on the same cell line. Gupta *et al.* identified three cell states (stem-like, basal-like and luminal-like cells) in SUM159 based on the three markers (EPCAM, CD24 and CD44). Based on prior knowledge, the stem-like cells were defined as CD44-high, CD24-neg, and EPCAM-low; basal-like cells were defined as CD44-high, CD24-neg and EPCAM-neg; and luminal-like cells were defined as CD44-low, CD24-high, and EPCAM-high. Figure 7C reproduces the Gupta *et al.* [3] gating strategy on FCM file analyzed in Figure 6. Gupta *et al.* strategy first gates the cells based on EPCAM high and low then gated the stem, luminal and basal like subpopulations based solely on CD24 low and high, as shown in Figure 7C. A cross classification table of our 9 subpopulations and the 3 Gutpa *et al.* cell states (labeled as stem, luminal and basal like subpopulations) is shown in Figure 7D. This analysis indicates that the basal-like subpopulation identified by the Gupta *et al.* gating is a combination of all the CCAST-derived cell states. Furthermore the analysis suggests that a mixture of basal-, stem- and luminal-cell like populations from the Gupta *et al.* sorting actually correspond to a single CCAST subgroup P9. This results implies that the cell-type specific analysis provided by Gupta *et al.* may have reflected the behavior of a single cell type. The Gupta *et al.* analysis may have been more informative if it were to investigate the distinct subpopulations, such as P1, P2, P5 and P7.

Finally, for experimental validation, we applied our CCAST-derived gating strategy on a SUM159 cell line in real time at a FACS machine. Supplemental Figure S3 shows the sorting result from this independent replicate; we are able to recover 5 distinct CCAST-derived subpopulations in real-time.

### CCAST differs from RchyOptimyx

We compare the application of CCAST and RchyOptimyx algorithm on the FCM data of the SUM159 breast cancer cell line. As briefly described in the Introduction, RchyOptimyx provides a gating strategy to identify target populations at various levels of purity [23]. On SUM159, RchyOptimyx initially generates 27 subpopulations for analysis. Because there is no clinical outcome variable to filter through these 27 predicted phenotypes using the RchyOptimyx algorithm, we selected only the phenotypes that correspond to a combination of CD24 and EPCAM for comparison to CCAST. Recall that CCAST resulted in 9 homogenous subpopulations that can be characterized in terms of these 2 markers alone. Based on use of EPCAM and CD24 alone RchyOptimyx yielded 12 subpopulations that can be targeted by a variety of gating strategies as shown in Supplement Figure S4. In other words, RchyOptimyx provides several possible paths to a particular subpopulation; in comparison, CCAST offers only a single path to target homogenous subpopulations thereby circumventing any additional interpretation of the output from RchyOptimyx for choosing the gating strategy. The underlying formalism of RchyOptimyx and CCAST are different but a full description of those differences is beyond the scope of this analysis.

## Discussion

We presented a model-based gating strategy, CCAST, for sorting a homogeneous subpopulation from a heterogeneous population of single cells without relying on expert knowledge. To identify a hierarchical 2D gating scheme to sort out homogeneous cells, we propose CCAST as a new approach that addresses three key and often-neglected questions: (1) How do we select the optimal markers for gating? (2) What is the optimal ordering of markers for sorting? (3) How do we estimate the marker cut offs for drawing the gates? The answers to these questions are usually decided in a subjective and bias manner making it very difficult to draw precise conclusions from the resulting sorted data. CCAST is an automated and unbiased strategy, requiring minimal human expertise, for optimizing gating of single cell data. While CCAST is optimized for cell sorting it can be applied for analysis of purified subpopulations among heterogeneous single cell data.

In all applications of CCAST in the study, we show that it is possible to characterize and isolate cell types based on a subset of the measured markers. When applied to normal bone marrow data, CCAST reveals an efficient strategy for gating T-cells. CCAST also produced an alternative gating framework for B-cells that produced a new characterization of mature B-cells into CD123+ and CD123- cells. The ability to isolate important cell subpopulations based on limited markers is particularly important since high-throughput cytometry technologies are increasing the number of markers they can measure and one will need new approaches to optimally select important set markers for gating. Hence CCAST not only provides the relevant marker set, optimized gating scheme, and reduces the need for human expertise, it can also reduce the number of antibodies needed for cell sorting.

We further motivated the need for CCAST as an automatically-generated gating scheme that does not rely on prior knowledge of cell states or marker relevance on the SUM159 breast cancer cell line. On this cell line Gutpa *et al.* tested the hypothesis that cancer cells can transition in any of the several possible cell states which exhibit important functional properties [3]. This study aimed to demonstrate the evidence of phenotypic switching between stem, basal and luminal breast cell states, which were defined by CD24 and EPCAM. Establishing strong evidence of cell state transitions would require pure cell states at onset, however, pure sorting is not evident by the manual gating scheme used in the study. In an independent study on the issue of phenotypic switching of cancer cell states, Zapperi and Porta [4] gave an alternative interpretation of the Gupta *et al.* based on an imperfect marker scenario. The CCAST analysis also infers nonhomogeneous subpopulations under the Gutpa *et al.* gating strategy and provided an alternative, more homogeneous cell states using an alternative gating strategy based on the same markers, namely CD24 and EPCAM. CCAST identifies at least 5 distinct breast cancer cell states in SUM159 and sorted out these pure cell states automatically (Figure 6) using only two surface markers, namely EPCAM and CD24. These subpopulations warrant further investigation to validate the notion of phenotypic switching in breast cancer cells as proposed by the Gupta *et al.* study.

CCAST enables the possibility to sort out unique underlying unknown cell states from a heterogeneous parent population in an optimal and unbiased manner using a gating scheme based on a decision tree representation. CCAST identifies homogeneous cell subpopulations using a non-parametric mixture distribution. Although several other clustering algorithms can also be used, CCAST can handle the unknown number of true clusters without the mathematical optimization of a distribution function. Silhouette coefficients are used to optimize the cell subpopulations and a recursive partitioning technique on the complete data given the cell states is used to generate the optimal decision tree for isolating the various subpopulations of interest. The partitioning comes after a marker selection step, which depends on a non-parametric test statistic making it completely data driven. CCAST also provides a confidence interval for marker cut-offs taking into account possible variability in marker distributions. For future methodological improvement on CCAST to both the computational cost and the pruning level L, one might consider multi way splits at each node, instead of using binary splits. Another methodological direction could be to use the confidence intervals to further enhance the decision trees; in particular, methods proposed by Katz *et al.* [25] can be adapted for CCAST.

In summary, CCAST is a fully automated model framework to identify a gating strategy to isolate subpopulations from single cell data with greater homogeneity compared to manual gating procedures. More generally, the CCAST framework could be used on other biological and non-biological high dimensional data types involving a mixture of unknown homogeneous subpopulations.

## Materials and Methods

### CCAST algorithm

CCAST formalizes the gating process of single cells as a statistical model and provides a simple unbiased hierarchical 2D gating scheme with the relevant set of marker cut-offs for gating a homogenous cell subpopulation given FCM data. Following, we describe the various steps in the non-parametric model framework of CCAST when applied to single cell data. A typical FCM dataset comprises simultaneous quantitative signal measurements of multiple biomarkers of single cells. These measurements can be fluorescence or atomic mass based. The data are stored in flow cytometry standard (FCS) files as a data frame with rows representing the cells or events and the columns corresponding to the markers of interest. Currently, we assume that the data have already been compensated to correct for spectral overlap during data generation and preprocessed using standard preprocessing steps in analysis of FCM data to remove spurious events. The data is then transformed using the recommended Arcsinh function [9] which can handle both positive and negative expression values.

#### CCAST applies non-parametric multivariate finite mixture models or hierarchical clustering for identifying cell subpopulations

The transformed FCM data is visualized as a high dimensional point cloud of cells, where each cell is a point in the cloud and each marker is represented as a single dimension in the cloud space. Different high-density cloud sub regions reflect abundance of specific cell subpopulations, which cannot be easily determined in high dimension. The first goal is to identify the cells that belong to the same cell subpopulation, that is cell type or state.

We propose a modification of a non-parametric multivariate mixture modeling approach by Benaglia *et al.* [19] for identifying homogeneous cell subpopulations among single cell data. This particular mixture model algorithm is an EM-like algorithm for non-parametric finite mixture modeling implemented in the mixtools R package [19]. It estimates the multivariate mixture distribution from multivariate random vectors. The vectors are assumed to have independent coordinates conditional upon knowing from which mixture component they come from, however, their density functions remain completely unspecified. The assignments of the random vectors to the most likely mixture component are done by maximizing aposterior probabilities. This algorithm is very flexible and can handle any number of mixture components and any number of vector coordinates of multivariate observations. Following [19] model specification and annotations, we denote the cell measurement vectors as 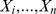 each comprising of 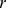 marker coordinates. We assume that they represent a sample from a finite mixture of 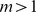 arbitrary distributions with each 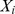 independent, conditional on the subpopulation 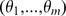 from which it is drawn. In addition, since some markers could be co-expressed in some cell subpopulations, we can also allow sets of markers to be identically distributed as well. Let 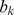 denote the set to which the 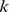 th marker belongs, where 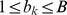 with 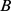 equal the total number of sets. The density of each 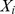 can be written as
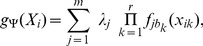
(1)where the function 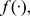 denotes an unknown univariate density function condition on the parameter space 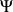 and 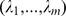 are all positive and sum to one. Thus for purpose of consistency, we use the indices 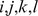 to denote a single unique cell, cell subpopulation, marker coordinate and marker set respectively. In general, we do not know apriori which marker sets define particular cell subpopulations. In this case we assume the more general model with 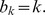 Equation 1 becomes



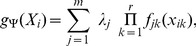
(2)Estimating all model parameters in Equation 1 is based on an EM-like algorithm [19] implemented in the mixtools R package using the “npEM” function. Similar to the EM Algorithm, this function defines a Bernoulli random variable 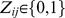 indicating that cell 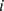 belongs to cell state 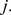 Hence 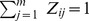 and the complete data becomes 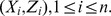 We first initialize all model parameters 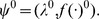 For every iteration 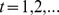

1. **E-step**: The posterior probabilities of cell assignment to a particular state condition on the data is given by,

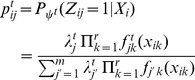
(3)

2. **M-step**: The proportion of cell states is given by,

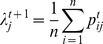
(4)for 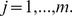

3. **KDE-step**: Given any real expression value 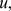 the non-parametric density estimate for some bandwidth parameter 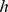 and kernel density function 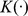 is given by,

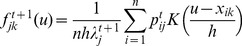
(5)The above algorithm is deterministic and in practice, the first iteration involves only an M-step with the 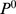 matrix obtained from a deterministic algorithm such as *k*-means clustering algorithm which assigns every cell a unique cell state. Note that the KDE-step depends on the kernel density 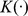 and a user defined bandwidth 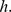 Following the recommendations by [19], the standard normal density function is used for 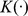 and a simple density dependent bandwidth rule given as
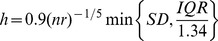
(6)is used, where 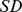 and 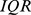 are the standard deviation and interquartile range of the pooled data. This method could either over estimate or under estimate the bandwidth. An updated iterative approach, which varies the bandwidth for each mixture component is also implemented in the mixtools R package. Note that any other clustering algorithm can also be used.

#### CCAST uses silhouette coefficients to refine cluster assignments

Another important issue involved in mixture modeling is selecting the number of components or in the context of FCM data, identifying the number of homogeneous cell subpopulations. We propose the initial use of a reasonable larger number of subpopulations than expected. The model provides the posterior distribution of cell subpopulation assignments whose MAP estimates in general result in a smaller number of cell subpopulation assignments corresponding to modes of high-density regions in the data space. Note that maximizing such a density function could result to wrong assignments of boundary cells to wrong cell states. Silhouette coefficients have been proposed as a diagnostic tool for evaluating the performance of clustering algorithms [26]. To improve on the sensitivity of the assignment of cells to their true subpopulation, we first estimate the silhouette coefficient 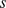 of all the cells. Starting from the initial 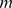 cluster assignments from the mixture model, cells with negative 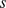 values are reassigned to the most likely neighbors. The memberships of cell subpopulations are updated and 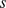 values associated with individual cells are recomputed. The same process is then repeated in the next iteration resulting in a new set of 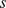 values, and so forth. This process converges to a stable number of cell subpopulations. We define a cost function by the absolute value of summing the negative silhouette values in each iteration. Minimizing the cost function optimizes the partition of the clusters. This process gives us a refined cell subpopulation distribution, which is required for generating the optimal gating scheme for the FCM data. Note that the silhouette step can be applied to any clustering output as well.

#### CCAST estimates a gating scheme for all cell subpopulations using a decision tree

Recursive partitioning is a well-established statistical technique that aims to correctly classify members of a certain population based on several dichotomous dependent variables in the form of a decision tree. This provides a decision rule for targeted classifications with more sensitivity or specificity. In the context of FCM data we formulate the gating process as a decision tree model with the vertices (nodes) corresponding to unknown markers of interest and the leaves of tree corresponding to the classification density of all the cell subpopulations. The root of the tree is the marker, which separates the cell subpopulations best. It has unique paths to all the leaves of the tree. A path gives a sequence of optimized rules leading to a given cell state subpopulation based on binary decisions on selected markers represented on the edges. The structure of the tree is determined by model based recursive partitioning technique by Horthon *et al.* [27]. This approach overcomes the variable selection bias and over-fitting problem, associated with most related techniques. A general framework of the algorithm works as follows:

1. **Step 1.** Test the global null hypothesis of independence between any of the continuous marker signal variables and the nominal response (which in this case is the distribution of the cell subpopulations from the mixture model described above). Stop if this hypothesis cannot be rejected. Otherwise select the marker signal with strongest association to the response. This association is measured by a p-value corresponding to a test for the partial null hypothesis of a single marker variable and the response.
2. **Step 2.** Implement a binary split in the selected marker variable.
3. **Step 3.** Recursively repeat steps 1) and 2).

One of such a framework has been implemented in the “ctree” function for conditional inference trees in the R party package [27] with a flexible stopping criteria of the derived tree. We go through the major steps above in more detail focusing mainly on the relevant information for implementation.

1. **Variable selection and stopping criteria.** Testing independence between a single marker variable and the derived cell states is equivalent to performing an association test between the nominal response variable 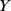 for cell states and an interval variable 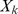 for each marker. The test statistic denoted as 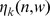 is constructed using conditional distribution of linear statistics in the permutation test framework [27], [28] based on both the permutation transformation of the data of size 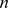 and associated weights 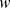 used in every node to build the tree. Each statistic 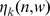 is used to generate a P-value 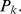 We select the marker 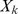 with minimum P-value. The stop criterion in step 1 in the main text is based on multiplicity adjusted p-values using multiple testing correction procedures. We reject the independence assumption when the minimum of the adjusted P-values is less than a pre-specified nominal level 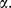 This parameter determines the size of the resulting tree.
2. **Splitting criteria.** Determining the optimal binary split point in the selected marker 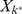 from the previous step is based on a goodness of split test using a two-sample linear statistics of the same form as in step 1 (see [28]). If we partition the selected marker space into all possible binary subsets 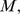 the optimal split 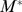 is derived from the maximum of all possible statistics associated with 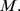 We control the number of subsets 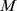 by restricting the sample size in each daughter node to some minimum value 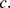

#### CCAST optimizes the decision tree by maximizing the size of the homogenous clusters

In practice the decision tree can be very large rendering its use almost impossible for manual gating. For practical purposes we propose the termination of the tree once we have identified all cell states as maximum in at least one of the leaf nodes during the partition process. This introduces a new parameter L, corresponding to a desired level of pruning. Note that this parameter will depend on the accuracy of the estimated cluster distribution of the data. We then perform the following;

1. **Step 1.** For each leaf node in the tree we select only the cells corresponding to the maximized cell state.
2. **Step 2.** Estimate a new decision tree on the updated data with maximum height L.
3. **Step 3.** Recursively repeat steps 1) and 2) until we get pure cell states at the leaf nodes.

This process provides a possible maximum sample size for all homogenous cell states simultaneously. Note that at the end it is possible to get more homogenous bins than expected. This could indicate the possibility of new clusters, which will require further confirmation. Also note that the removed data points during the updating process can also be put into an extra bin for further investigation.

#### CCAST is available as an R package

CCAST algorithm has been implemented as an R package with examples and documentation. It is available as a zip file in the supplementary information.

### FACS analysis of SUM159

Sum 159 cells were cultured in Ham F12 medium supplemented with 5% calf serum, insulin (5 ug/ml), hydrocortisone, Pen/Strep/L-Glutamine. Cells were grown at 37° C in a 5% CO2 incubator. Stock aliquots of cells were frozen in 10% DMSO and 90% FBS and stored in −80° C liquid nitrogen. The cells were thawed initially into T25 flasks and allowed to expand in culture for two weeks prior to sorting (expanded into T75 flasks). The day of sort, cells were trypsinized, washed with PBS and stained with antibodies specific for the following human cell surface markers: EPCAM (ESA)-FITC (AbD Serotec, MCA1870F), CD24-PE (BD Biosciences), CD44-APC (BD Biosciences), CD49f-PerCP/Cy5.5 (Biolegend). Roughly 1×107 cells were incubated with antibody (20uL antibody per million cells) for 15 min at room temperature in PBS with 1% BSA. Unbound antibody was washed off and cells were analyzed on a custom Stanford and Cytek upgraded FACScan (Beckman Center, Stanford) no more than one hour after staining. Cell sorting was performed on BD Aria II (Beckman Center, Stanford). The raw data is available in supplement Dataset S1 as an FCS file.

## Supporting Information

Algorithm S1 **CCAST algorithm implemented as an R package.** The algorithm, named CCAST for Clustering, Classification and Sorting Tree, identifies and isolates homogeneous cell subpopulations from heterogenous single cell data in an optimal and unbiased manner using a decision tree representation that can be applied to cell sorting and data analysis.
(GZ) Click here for additional data file.

Dataset S1 **FCM data for SUM159 breast cancer cell line.** Single cell data for SUM159 breast cancer cell line stored as an FCS file.
(ZIP)

Figure S1 **CCAST decision tree height (L) analysis on training data.** **A** The CCAST gating strategy based on the unlabeled T-cell training data shows exactly the same decision tree as in Figure 3A after increasing L to 3 or more levels. **B** The CCAST gating strategy based on the unlabeled T-cell test data shows that all split point estimates lie within the estimated confidence intervals shown in Figure 3A derived from the training data.
(TIF)

Figure S2 **CCAST gating strategy on SUM159 breast cancer cell line in flowJo.** The implementation of the CCAST gating strategy based on SUM159 breast cancer cells using flowJo showing 9 homogeneous clusters.
(TIF)

Figure S3 **SUM159 breast cancer cell analyzed on FACS machine in real-time.** Top panel: CCAST-derived unique five subpopulations, labeled as P1 thru P5 using gating strategy in Figure 6. Bottom panel: Proof that the CCAST-derived gating scheme in Figure 6 works on an independent real-time sort of populations P1 thru P5. See Materials and Methods for experimental details.
(TIF)

Figure S4 **RchyOptimyx analysis on breast cancer cell line.** The implementation of the RchyOptimyx tool on SUM159 Breast cancer cell line yielded 12 subpopulations defined on EPCAM and CD24. These populations can be targeted by a variety of gating strategies illustrated here as Strategy 1-12.
(TIF)

Table S1 **Simulated single cell data for CCAST.** We simulated 850 cell expression measurements on 3 markers from a mixture of 5 states whose global expression pattern depict cell state progression. Celltype 1 is characterized as “low”, “low”, “high”. Celltype 2 is characterized as “high low”, “low mid”, “high”, Celltype 3 is characterized as “mid”, “mid”, “high”, Celltype 4 is characterized as “low high”, “low high”, “high” and Celltype 5 is characterized as “high”, “high”, “high”. We use different normal distributions to quantify these cell states.
(TIF)
