# Supplementary material for: CCAST: A Model-Based Gating Strategy to Isolate Homogeneous Subpopulations in a Heterogeneous Population of Single Cells
Source: PLoS Comput Biol. 2014 Jul 31;10(7):e1003664. doi: 10.1371/journal.pcbi.1003664 (PMC4117418; doi:10.1371/journal.pcbi.1003664)
Supplement: Algorithm S1 — CCAST algorithm implemented as an R package. The algorithm, named CCAST for Clustering, Classification and Sorting Tree, identifies and isolates homogeneous cell subpopulations from heterogenous single cell data in an optimal and unbiased manner using a decision tree representation that can be applied to cell sorting and data analysis. (GZ) [file pcbi.1003664.s001.gz › CCAST/inst/doc/ccast.pdf]

—CCAST : A model-based gating strategy— Examples in *T and Breast cancer* single cells

Benedict Anchang*

May 23, 2014

Abstract

In single cell analysis, one often needs a simple hierarchical 2D gating scheme to isolate homogeneous live cells for further downstream analysis. Most automated gating algorithms for single cell data developed so far can be used to identify subpopulations of interest in high dimensional space across different samples. However, these approaches still require some expert knowledge to isolate these subpopulations manually for downstream analysis that may not be optimal, particularly for single cell data whose clusters in high dimensional space are very close to each other and have regions were there is a mixture of cell states such as tumor cancer single cell data. We propose CCAST as a new approach that addresses three key and often-neglected questions: (1) How do we select the optimal markers for gating? (2) What is the optimal ordering of markers for sorting? (3) How do we estimate the marker cut offs for drawing the gates? The answers to these questions are usually decided in a subjective and bias manner making it very difficult to draw precise conclusions from the data. We propose CCAST as a new sorting strategy that is automated and unbiased, requiring minimal human expertise for optimizing gating of single cell data and provide its usage in two applications: bone marrow T and breast cancer single cells.

CCAST takes as input a file path or directory path name with all fcs files of interest or a cxm flow cytometry(FCM) or mass cytometry(MCM) expression matrix of c number of cells and m number of markers. The output is a list comprising (1) an initial decision cast tree showing the optimal pruning level for optimizing the size of the various homogeneous subgroups, (2) a final decision tree with unimodal leaf nodes, (3) a confidence limits for all inner nodes cut off variables if required. In addition, CCAST produces several pdf files corresponding

*Department of Radiology, Center for Cancer Systems Biology, Stanford University, Stanford, CA, 94305-5488, USA ; URL: http://plevritis.stanford.edu/people.html

to various diagnostic plots of the predicted homogenous sub populations as well as biaxial scatter plots for all markers identified by CCAST used to build the final decision tree showing the marker cut-offs. These plots represent a visual reproducible gating scheme that can be applied to an independent replicate sample in real time.

1 Introduction

This document demonstrates the functionality of the CCAST package by providing a gating strategy for FCM and MCM assays using a decision tree representation. CCAST identifies homogeneous cell subpopulations using a non-parametric mixture distribution or hierarchical clustering. Any other clustering algorithms can also be used. Silhouette coefficients are used to optimize the cell subpopulations and a recursive partitioning technique on the complete data given the cell states is used to generate the optimal decision tree for isolating the various subpopulations of interest. The partitioning comes after a marker selection step, which depends on a non-parametric test statistic making it completely data driven. CCAST also provides a confidence interval for marker cut-offs taking into account possible variability in marker distributions [1].

2 Installation

CCAST relies on R libraries: mixtools (>=1.0.0), flowCore (>= 1.11.20), spade (>=1.10.2), fastcluster (>= 1.5), cluster(>= 1.14.4), ggplot2 (>= 0.9.3.1), scales (>= 0.2.3), RBGL (>= 1.8.1), party (>= 1.0-10), RColorBrewer (>=1.0-5), car(>= 2.0-19). To install the package open a shell and go to the directory containing the CCAST package. The package can be installed using the following commands:

```
shell$ R CMD INSTALL -l /path/to/library CCAST  ## within program console
>install.packages(/path/to/library CCAST,repos = NULL, type="source") ## within R
```

3 Loading the Library

We start by loading the library (for installation guidelines see the Bioconductor website). Next create a working directory (cast output path) to save all CCAST output figures and data.

```
>library(CCAST)
>dir.create(path/to/CCAST output)
```

4 Major steps in CCAST

CCAST formalizes the gating process of single cells as a statistical model using a clustering approach and silhouette measures to quantify underlying homogeneous subpopulations of cells, then recursive partitioning techniques to generate the decision tree comprising the gating strategy for all subpopulations of interest. The output is a simple unbiased hierarchical 2D gating scheme with the relevant set of marker cut-offs for gating a homogenous cell subpopulations from given FCM data.

4.1 CCAST applies non-parametric multivariate finite mixture models or hierarchical clustering for identifying cell subpopulations

CCAST makes use of non-parametric mixtures or hierarchical clustering for subpopulation identification.The former model based approach is an EM-like algorithm for non-parametric finite mixture modeling implemented in the mixtools R package [2] using the "npEM" function. It estimates the multivariate mixture distribution from multivariate random vectors. The vectors are assumed to have independent coordinates conditional upon knowing from which mixture component they come from, however, their density functions remain completely unspecified. The assignments of the random vectors to the most likely mixture component are done by maximizing aposterior probabilities. This approach is time consuming for very large data. CCAST automatically uses the hierarchical clustering approach developed in the fastcluster R package [3] once the data has more than 3000 events. CCAST also uses silhouette coefficients to refine the derived clusters. The silhouette step has been made optional inorder to speed up the algorithm and has been implemented only for data with sample size less than or equal to 30000.

4.2 CCAST estimates an initial gating scheme for all cell states using a decision tree

CCAST formulates the gating process as a decision tree model with the vertices (nodes) corresponding to unknown markers of interest and the leaves of the tree corresponding to the classification density of all the cell subpopulations. The root of the tree is the marker, which separates the cell

subpopulations best. It has unique paths to all the leaves of the tree. A path gives a sequence of optimized rules leading to a given cell state subpopulation based on binary decisions on selected markers represented on the edges. The structure of tree is determined by model based recursive partitioning technique by Horthon *et al.* [4]. This approach overcomes the variable selection bias and overfitting problem, associated with most related techniques.

4.3 CCAST optimizes the decision tree by maximizing the size of the homogenous clusters

In practice the decision tree can be very large rendering its use almost impossible for manual gating. For practical purposes CCAST terminates the tree once all cell states have been identified as maximum in atleast one of the leaf nodes during the partition process. This introduces a new parameter L, corresponding to a desired level of prunning. CCAST updates the tree by removing misclassified events in the leaf nodes and reestimates the tree until there is no misclassification error.

4.4 CCAST implements a bootstrap analysis to assess the range of values for the split points in the optimal decision tree

CCAST uses a strata-sampling method with replacement to generate B bootstrap datasets of the same sample size. It then generates B decision trees with different split points and estimates confidence intervals of the split points by minimum and maximum boundary estimates from the bootstrap estimates.

5 CCAST provides an efficient gating strategy for T-cells

We first demonstrate the applicability of CCAST on real FCM hematopoietic dataset with about 10000 cells, using single cell data from a study by Bendall *et al.* [6] of normal bone marrow based on mass cytometry, a recently developed high throughput technology for labeling cells with no auto fluorescence effect. An appeal of this study is that hematopoietic cells have a well-established differentiation process and set of lineage markers. In this study, unstimulated human peripheral blood mononuclear cells (PBMCs) from a healthy donor were analyzed using thirteen surface parameters from

a 34-parameter mass cytometry data. The thirteen surface antigens measured include: CD45, CD45RA, CD19, CD11b, CD4, CD8, CD34, CD20, CD33, CD123, CD38, CD90, and CD3. The manual gating process and the characterization of the major cell populations are shown in Figure S5 of the supplement material from [6]. The study identified two subsets, among many, of cells involved in both T-cell development. The T subset included naive CD4 and CD8 T-cells and mature CD4 and CD8 T-cells. For this analysis we pooled all the cells.

```
>data(Dall)
>biomarker=c("CD45","CD45RA","CD19","CD11b","CD4","CD8",
"CD34","CD20","CD33","CD123","CD38","CD90","CD3")
>colnames(Dall)=biomarker
>colid=1:dim(Dall)[2]
>Result1<-ccast_main(file=Dall, transformlogic=FALSE, asinhp=1, deterministic=TRUE,
 colid, coln=NULL, rown=NULL, npmix=FALSE, k=4, boot=NULL, ylabel="CD8",
origscale=FALSE, groups=NULL,runsilhouette=FALSE)
```

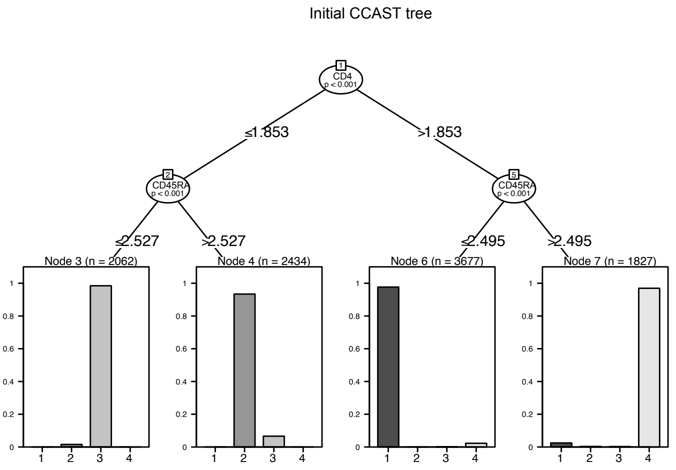

Figure 1: **T cell analysis. A** Initial Gating strategy for T cell types based on CD45 and CD45RA markers.

Figure 1 and Figure 2 corresponding to the intial anfd final CCAST trees show that the 4 distinct T cell states can easily be isolated using only 2 of the 13 measured surfaces markers, namely CD45 and CD45RA. This result demonstrates that CCAST produces an optimal gating strategy even with a very high dimensional FCM data because it found that it is still possible to extract pure cell states using a relatively small number of markers. Figure 3

shows a 2D scatter plot of the 2 markers that partition the data into clearly 4 clusters comparing for both the original data and the final CCAST purified data. Although there is a strong evidence of 4 clusters (Figure 3 A), it would be very challenging to gate out all the population in purple without contaminating these cells with those color coded in red. Note all foreign points mixed with various clusters have been removed from the purified data (Figure 3 B). For manual gating purposes comparing the two schemes **A** and **B** provides a visual confidence on how to gate out more pure subpopulations.

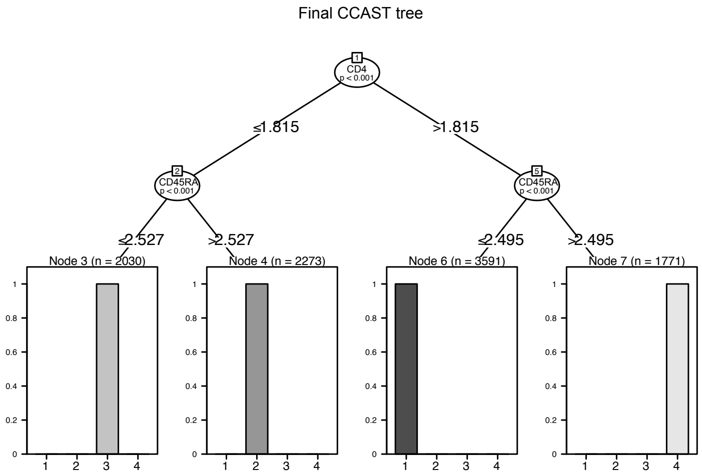

Figure 2: **T cell analysis.** Optimal gating strategy for T cell types based on CD45 and CD45RA markers.

6 CCAST identifies at least 5 cell states in SUM159 breast cancer cell line

We next show the usage of CCAST as a completely automated gating scheme with no prior knowledge of cell states or marker relevance on about 11000 breast cancer cells from SUM159 cell line. based on a study by [7]. Gutpa *et al.* explored the hypothesis that cancer cells can transition in any of the several possible phenotypic states which exhibit important functional properties [7]. Gutpa *et al.* used the SUM159 breast cancer cell line to demonstrate the evidence of phenotypic switching between stem, basal and luminal breast cell states, which were defined by EPCAM, CD24 and CD44. Although we assumed a prior number of 5 clusters, CCAST identifies more than 5 cell states in SUM159 breast cancer cell line defined by EPCAM and

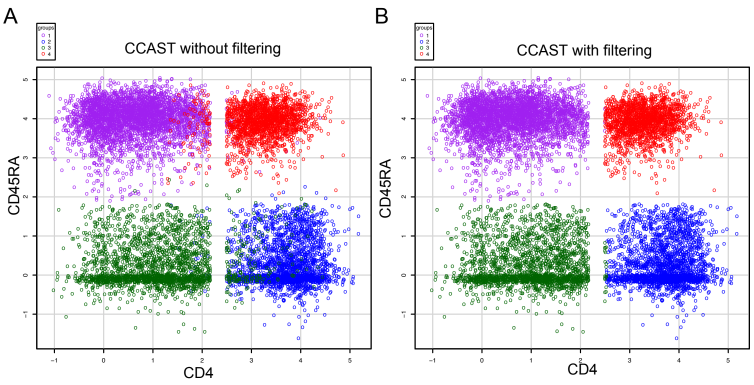

Figure 3: **T cell analysis.** **A** 2D scatter plot for all data showing prior 4 clusters color coded as purple, blue, green, and red. Note manual gating the population in purple and blue will likely result in contamination of points in red and green even if there are apparently four clusters. **B** 2D scatter plot from CCAST based on CD45 and CD45RA showing the 4 pure cell state populations. Note all foreign points mixed with various clusters from **A** have been removed. For manual gating purposes comparing the two schemes **A** and **B** provides a visual confidence on how to gate out pure subpopulations.

CD24 alone. Establishing strong evidence of cell state transitions would require pure cell states at onset, which is not evident by the manual gating scheme used in the study.

```
>data(sum159)
>colid=1:dim(Dall)[2]
>Dall2=asinh(Dall)
>Result2<-ccast_main(file=Dall2, transformlogic=FALSE, asinhp=1, deterministic=TRUE,
colid, coln=NULL, rown=NULL, npmix=FALSE, k=5, boot=NULL, ylabel="CD44",
origscale=TRUE, groups=NULL,runsilhouette=TRUE)
```

The final CCAST output from the CCAST gating strategy applied on a subset of about 11000 breast cancer cells is shown in Figure 4. Bar plots which are one of the diagnostic plots from CCAST corresponding to all 7 subgroups at the leaf nodes of the final tree with standard deviation intervals for each marker is shown in Figure 5.

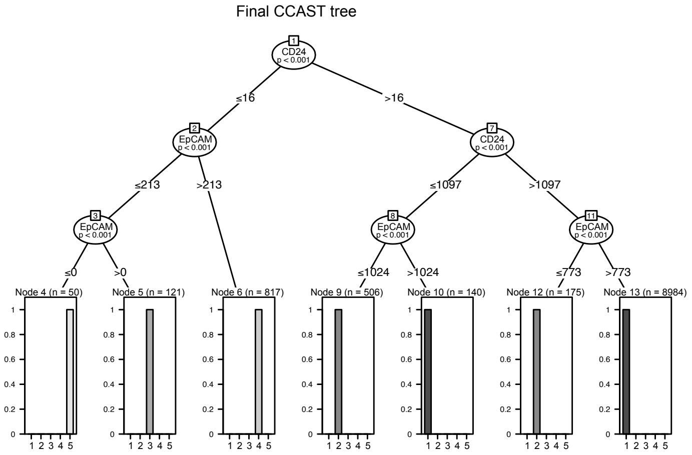

Figure 4: **Breast cancer results.** Optimal gating strategy for SUM159 breast cancer cell lines isolates 5 or more pure cell states(7 bins) mainly based on CD24 and EPCAM.

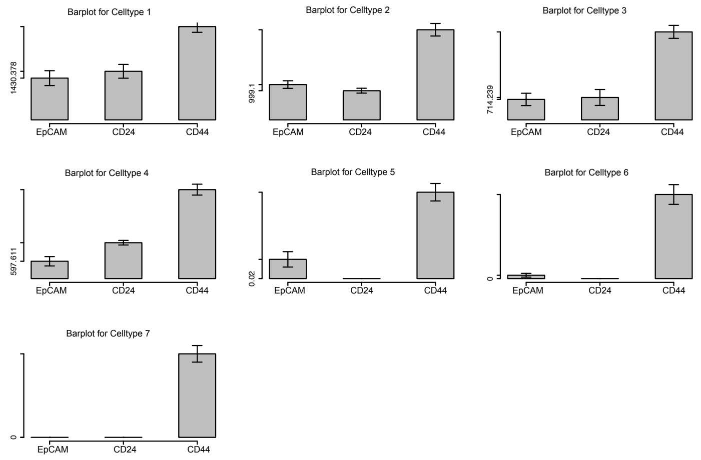

Figure 5: **Breast cancer results.** Bar plots of the 7 homogenous bins from Figure 4 across all 3 markers with standard deviation intervals for each marker. The values on the bars on the left side of each plot correspond to the minimum value for all 3 bar heights. Each side bar gives a sense of the relative difference between bar heights. Each labeled subpopulation corresponds to one of the leaf node bins in the tree in Figure 4.

References

[1] Anchang, B , et al. (2014) CCAST: A model-based gating strategy to isolate homogeneous cell subpopulations in heterogeneous single cell data, *Plos Computational Biology.*

[2] Benaglia, T, Chauveau, D and Hunter, D R (2009) An EM-like algorithm for semi- and non-parametric estimation in multivariate mixtures, *Journal of Computational and Graphical Statistics* 18:505-526.

[3] Mullner, D. (2013) fastcluster: Fast Hierarchical, Agglomerative Clustering Routines for R and Python.*Journal of Statistical Software* 53(9):1-18.

[4] Hothorn, T, Hornik, K and Zeileis, A (2006) Unbiased recursive partitioning : A conditional inference framework, *Journal of Computational and Graphical Statistics* 15: 651-674.

[5] Rousseeuw, P J (1987) Silhouettes: A graphical aid to the interpretation and validation of cluster analysis. *Journal of Computational and Applied Mathematics* 20:53-65

[6] Bendall, S C *et al.* (2011) Single Cell Mass Cytometry of Differential Immune and Drug Responses Across the Human Hematopoietic Continuum. *Science* 332(6030):687-696.

[7] Gupta, P B, Fillmore, C M, Jiang, G, Shapira,S D, Tao, K, Kuperwasser, C and Lander, E S (2011) Stochastic State Transitions Give Rise to Phenotypic Equilibrium in Populations of Cancer Cells, *Cell* 146:633-644.
