# Supplementary figures and images for: CCAST: A Model-Based Gating Strategy to Isolate Homogeneous Subpopulations in a Heterogeneous Population of Single Cells
Source: PLoS Comput Biol. 2014 Jul 31;10(7):e1003664. doi: 10.1371/journal.pcbi.1003664 (PMC4117418; doi:10.1371/journal.pcbi.1003664)

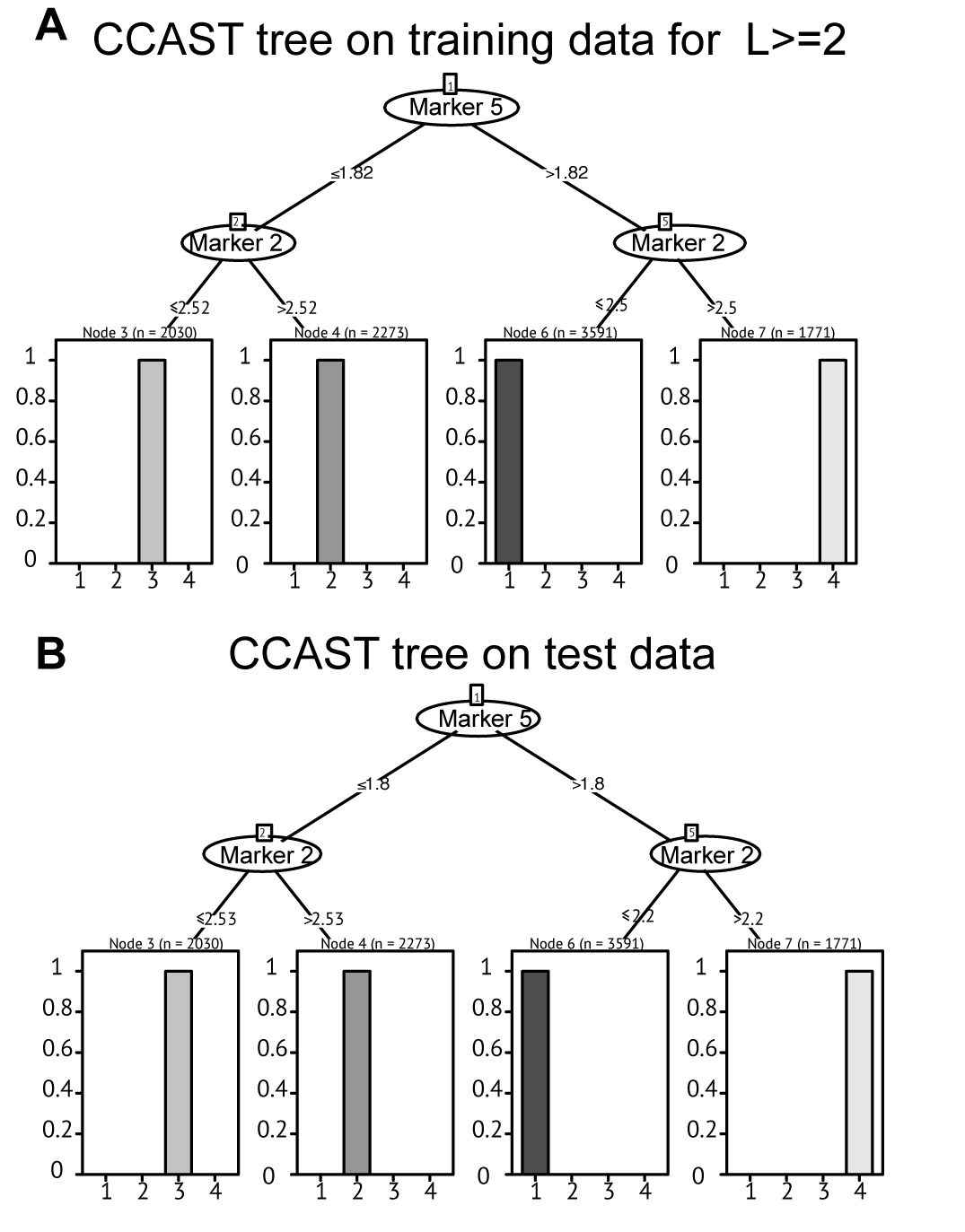

Supplement: Figure S1 — CCAST decision tree height (L) analysis on training data. A The CCAST gating strategy based on the unlabeled T-cell training data shows exactly the same decision tree as in Figure 3A after increasing L to 3 or more levels. B The CCAST gating strategy based on the unlabeled T-cell test data shows that all split point estimates lie within the estimated confidence intervals shown in Figure 3A derived from the training data. (TIF) [file pcbi.1003664.s003.tif]

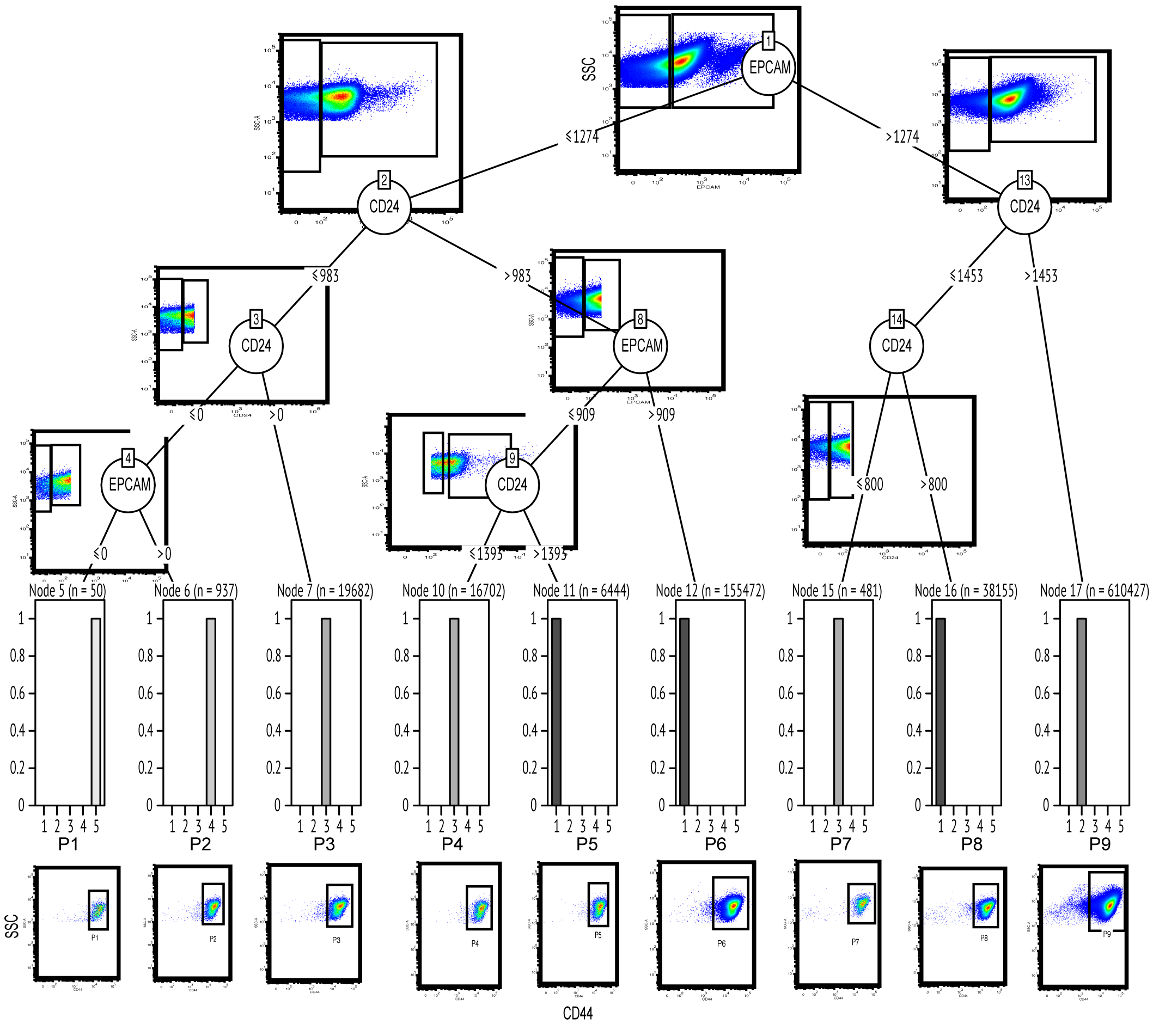

Supplement: Figure S2 — CCAST gating strategy on SUM159 breast cancer cell line in flowJo. The implementation of the CCAST gating strategy based on SUM159 breast cancer cells using flowJo showing 9 homogeneous clusters. (TIF) [file pcbi.1003664.s004.tif]

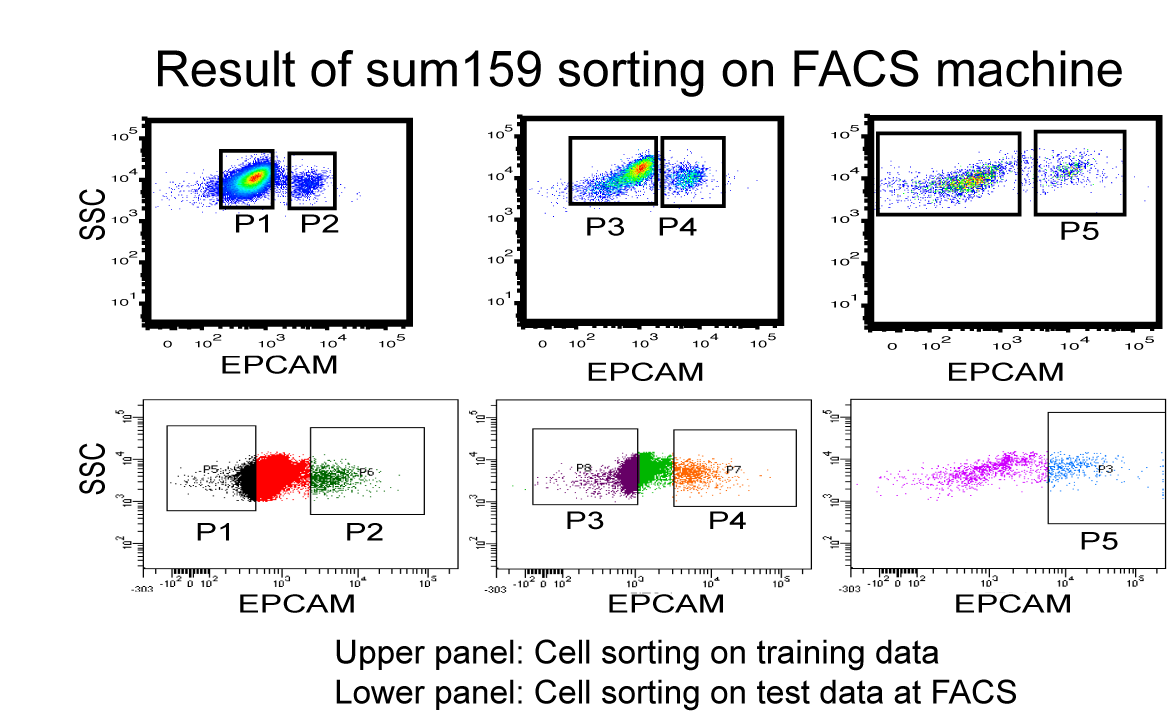

Supplement: Figure S3 — SUM159 breast cancer cell analyzed on FACS machine in real-time. Top panel: CCAST-derived unique five subpopulations, labeled as P1 thru P5 using gating strategy in Figure 6. Bottom panel: Proof that the CCAST-derived gating scheme in Figure 6 works on an independent real-time sort of populations P1 thru P5. See Materials and Methods for experimental details. (TIF) [file pcbi.1003664.s005.tif]

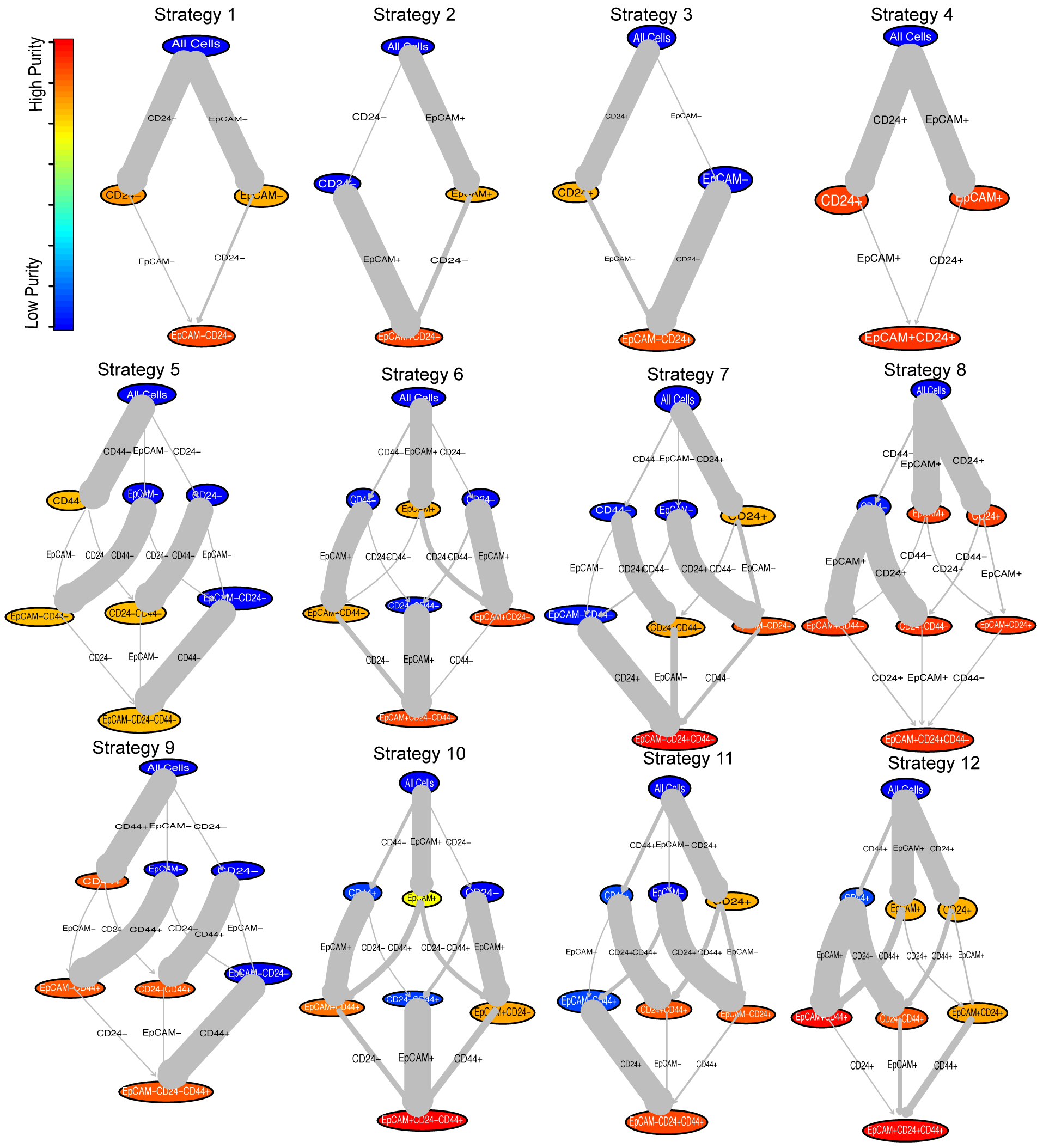

Supplement: Figure S4 — RchyOptimyx analysis on breast cancer cell line. The implementation of the RchyOptimyx tool on SUM159 Breast cancer cell line yielded 12 subpopulations defined on EPCAM and CD24. These populations can be targeted by a variety of gating strategies illustrated here as Strategy 1-12. (TIF) [file pcbi.1003664.s006.tif]

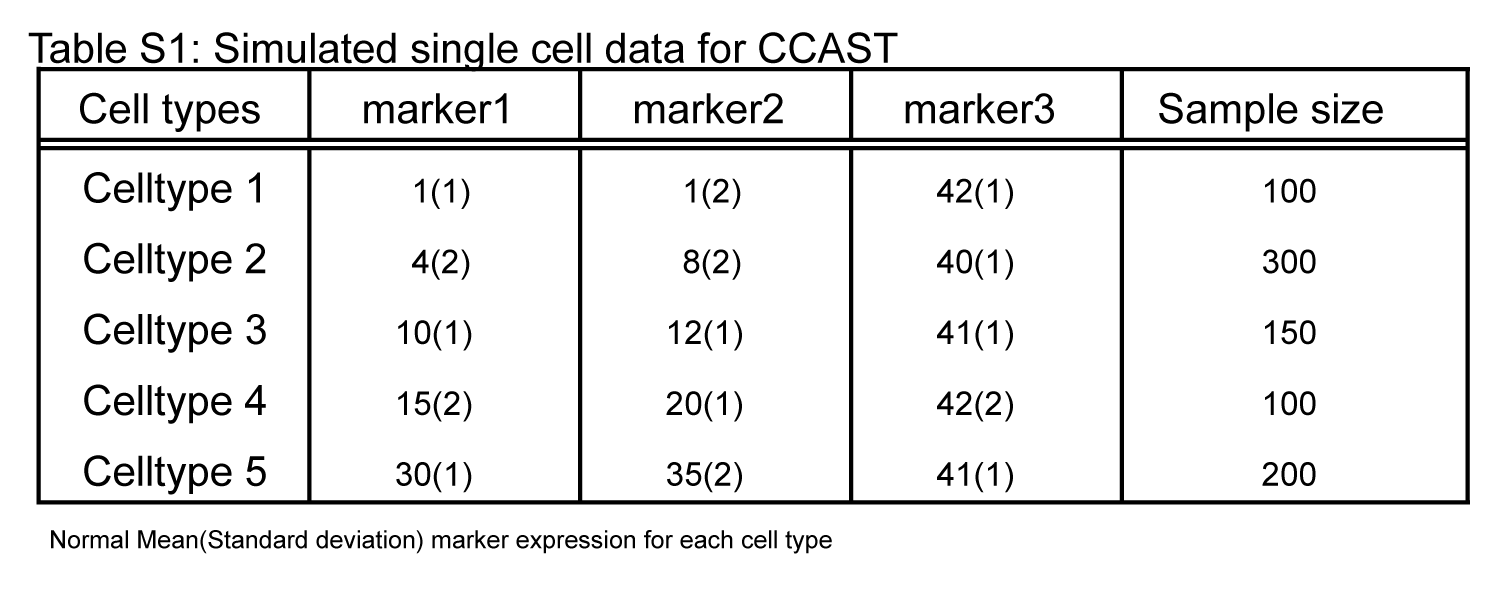

Supplement: Table S1 — Simulated single cell data for CCAST. We simulated 850 cell expression measurements on 3 markers from a mixture of 5 states whose global expression pattern depict cell state progression. Celltype 1 is characterized as “low”, “low”, “high”. Celltype 2 is characterized as “high low”, “low mid”, “high”, Celltype 3 is characterized as “mid”, “mid”, “high”, Celltype 4 is characterized as “low high”, “low high”, “high” and Celltype 5 is characterized as “high”, “high”, “high”. We use different normal distributions to quantify these cell states. (TIF) [file pcbi.1003664.s007.tif]
